# GWAS Meta-analysis of Kidney Function Traits in Japanese Populations

**DOI:** 10.2188/jea.JE20230281

**Published:** 2024-11-05

**Authors:** Asahi Hishida, Masahiro Nakatochi, Yoichi Sutoh, Shiori Nakano, Yukihide Momozawa, Akira Narita, Kozo Tanno, Atsushi Shimizu, Atsushi Hozawa, Kengo Kinoshita, Taiki Yamaji, Atsushi Goto, Mitsuhiko Noda, Norie Sawada, Hiroaki Ikezaki, Mako Nagayoshi, Megumi Hara, Sadao Suzuki, Teruhide Koyama, Chihaya Koriyama, Sakurako Katsuura-Kamano, Aya Kadota, Kiyonori Kuriki, Masayuki Yamamoto, Makoto Sasaki, Motoki Iwasaki, Keitaro Matsuo, Kenji Wakai

**Affiliations:** 1 Department of Public Health, Aichi Medical University School of Medicine, Aichi, Japan; 2 Department of Preventive Medicine, Nagoya University Graduate School of Medicine, Aichi, Japan; 3 Public Health Informatics Unit, Department of Integrated Health Sciences, Nagoya University Graduate School of Medicine, Aichi, Japan; 4 Division of Biomedical Information Analysis, Iwate Tohoku Medical Megabank Organization, Iwate, Japan; 5 Division of Biomedical Information Analysis, Iwate Medical University, Iwate, Japan; 6 Division of Epidemiology, National Cancer Center Institute for Cancer Control, Tokyo, Japan; 7 Laboratory for Genotyping Development, Center for Integrative Medical Sciences, RIKEN, Kanagawa, Japan; 8 Tohoku Medical Megabank Organization, Tohoku University, Miyagi, Japan; 9 Division of Clinical Research and Epidemiology, Iwate Medical University, Iwate, Japan; 10 Department of Hygiene and Preventive Medicine, Iwate Medical University, Iwate, Japan; 11 Department of Public Health, School of Medicine, Yokohama City University, Kanagawa, Japan; 12 Department of Diabetes, Metabolism and Endocrinology, Ichikawa Hospital, International University of Health and Welfare, Chiba, Japan; 13 Department of Endocrinology and Diabetes, Saitama Medical University, Saitama, Japan; 14 Division of Cohort Research, National Cancer Center Institute for Cancer Control, Tokyo, Japan; 15 Department of Comprehensive General Internal Medicine, Graduate School of Medical Sciences, Kyushu University, Kyushu University Hospital, Fukuoka, Japan; 16 Department of General Internal Medicine, Kyushu University Hospital, Fukuoka, Japan; 17 Department of Preventive Medicine, Faculty of Medicine, Saga University, Saga, Japan; 18 Department of Public Health, Nagoya City University Graduate School of Medical Sciences, Aichi, Japan; 19 Department of Epidemiology for Community Health and Medicine, Kyoto Prefectural University of Medicine, Kyoto, Japan; 20 Department of Epidemiology and Preventive Medicine, Kagoshima University Graduate School of Medical and Dental Sciences, Kagoshima, Japan; 21 Department of Preventive Medicine, Tokushima University Graduate School of Biomedical Sciences, Tokushima, Japan; 22 Center for Epidemiologic Research in Asia, Shiga University of Medical Science, Shiga, Japan; 23 Laboratory of Public Health, Division of Nutritional Sciences, School of Food and Nutritional Sciences, University of Shizuoka, Shizuoka, Japan; 24 Iwate Tohoku Medical Megabank Organization, Disaster Reconstruction Center, Iwate Medical University, Iwate, Japan; 25 Division of Ultrahigh Field MRI, Institute for Biomedical Sciences, Iwate Medical University, Iwate, Japan; 26 Division of Cancer Epidemiology and Prevention, Aichi Cancer Center Research Institute, Aichi, Japan; 27 Department of Cancer Epidemiology, Nagoya University Graduate School of Medicine, Aichi, Japan

**Keywords:** genome-wide association study, serum creatinine, estimated glomerular filtration rate, chronic kidney disease, cluster of differentiation 36

## Abstract

**Background:**

Genetic epidemiological evidence for the kidney function traits in East Asian populations, including Japanese, remain still relatively unclarified. Especially, the number of genome-wide association studies (GWASs) for kidney traits reported still remains limited, and the sample size of each independent study is relatively small. Given the genetic variability between ancestries/ethnicities, implementation of GWAS with sufficiently large sample sizes in specific population of Japanese is considered meaningful.

**Methods:**

We conducted the GWAS meta-analyses of kidney traits by leveraging the GWAS summary data of the representative large genome cohort studies with about 200,000 Japanese participants (*n* = 202,406 for estimated glomerular filtration rate [eGFR] and *n* = 200,845 for serum creatinine [SCr]).

**Results:**

In the present GWAS meta-analysis, we identified 110 loci with 169 variants significantly associated with eGFR (on chromosomes 1–13 and 15–22; *P* < 5 × 10^−8^), whereas we also identified 112 loci with 176 variants significantly associated with SCr (on chromosomes 1–22; *P* < 5 × 10^−8^), of which one locus (more than 1 Mb distant from known loci) with one variant (*CD36* rs146148222 on chromosome 7) for SCr was considered as the truly novel finding.

**Conclusion:**

The present GWAS meta-analysis of the largest genome cohort studies in Japanese subjects provided some original genomic loci associated with kidney function, which may contribute to the possible development of personalized prevention of kidney diseases based on genomic information in the near future.

## INTRODUCTION

Chronic kidney disease (CKD) is a growing public health burden worldwide, as well as in East Asian countries. Progression of CKD leads to kidney failure, which eventually requires hemodialysis and kidney transplantation, and finally causes cardiovascular disease (CVD) mortality, especially if not adequately treated.^1^ Meanwhile, recent achievements in genetic epidemiological studies discovered genomic loci associated with human kidney functions.^2^^–^^4^ The worldwide-scaled genome-wide association study (GWAS) consortium, the Chronic Kidney Disease Genetics Consortium (CKDGen) revealed 424 estimated glomerular filtration rate (eGFR) loci based on the investigation of 1.2 million individuals.^5^

However, the genetic epidemiological evidence for the kidney function traits in East Asian populations, including Japanese, remain relatively unclarified. Especially, the number of GWASs for kidney traits reported are still limited, as represented by the GWAS meta-analysis of kidney traits in the East Asians and GWAS of kidney function traits using the data of the Japan Multi-Institutional Collaborative Cohort (J-MICC) Study, a large genome cohort study consisted of about 11,000 participants,^3^^,^^4^ and the sample size of each independent study is relatively small. Given the genetic variations between ancestries/ethnicities, implementation of GWAS with sufficiently large sample sizes in the specific Japanese population is considered meaningful, which may provide clues for the possible preventive measures against CKD development in the ageing society of Japan. Accordingly, we conducted the GWAS meta-analyses of kidney traits by leveraging the GWAS summary data of the representative large genome cohort studies in Japan.

## METHODS

### Study participants

#### J-MICC

The J-MICC Study was launched in 2005 in 10 areas of Japan, in which about 100,000 volunteers aged 35–69 years provided their blood and lifestyle data based on a questionnaire, after providing informed consent.^6^^–^^8^ GWAS genotyping was performed in 14,539 randomly selected study participants from 12 areas (Chiba, Sakuragaoka, Shizuoka-Daiko, Okazaki, Aichi, Takashima, Kyoto, Tokushima, Fukuoka, Saga, Kagoshima and Kyushu-KOPS [Kyushu Okinawa Population Study]) where the J-MICC Study took place. Thereafter, data from 11,268 participants from 10 areas (Sakuragaoka, Shizuoka-Daiko, Okazaki, Takashima, Kyoto, Tokushima, Fukuoka, Saga, Kagoshima and Kyushu-KOPS) who had their serum creatinine (SCr) levels measured with the enzymatic method or with the Jaffe method (whose creatinine levels were converted to those in enzymatic method) were provided for the analyses. The J-MICC Study protocol was approved by the ethics committee of Nagoya University Graduate School of Medicine (approval no.: 253) and by those of the affiliated institutions.

#### JPHC

The Japan Public Health Center-based Prospective (JPHC) Study recruited residents aged 40–69 years from 11 public health centers in 1990 (cohort I) and 1993–1994 (cohort II) and conducted four questionnaire surveys at 5-year intervals.^9^ Among a subsample of the JPHC study participants who received health check-ups, we carried out two diabetes surveys that asked additional questionnaires about diabetes and collected clinical data including SCr levels measured at the health check-ups, in 1998–1999 (the first diabetes survey) or 2003–2004 (the second diabetes survey) for cohort II and 2000 (the first diabetes survey) or 2005 (the second diabetes survey) for cohort I.^10^ In JPHC1, after excluding residents in two public health centers because of different inclusion criteria, participants for genetic research were randomly drawn from 16,983 residents who provided their questionnaires and 10 mL of venous blood at the time of the first questionnaire survey and participated in the first diabetes survey. Consequently, 6,616 participants were identified for genotyping. In JPHC2, after excluding residents in two public health centers because of different inclusion criteria, participants for genetic research were randomly drawn from 7,229 residents who provided their questionnaires and 10 mL of venous blood at the time of the second questionnaire survey, participated in the first diabetes survey, and were not included in the JPHC1 study. Consequently, 2,630 participants were identified for genotyping. Before performing genetic research in JPHC1 and JPHC2, we obtained approval from the institutional review board of the National Cancer Centre (Approval No.: 2011-044), Tokyo, Japan, and provided eligible participants with the option of refusing participation.

#### TMM

The Tohoku Medical Megabank Community-Based Cohort (TMM CommCohort) study was described previously.^11^ In brief, 20- to 75-year-old residents from Iwate and Miyagi, the Pacific coast prefectures in Northeast Japan, were recruited between May 2013 and March 2016. The present study included 53,599 participants genotyped using Japonica array version 2. The protocol of TMM CommCohort Study was approved by Ethical Committee of ToMMo (the first approval no.: 2012-4-617 and the latest approval no.: 2018-4-087) and the Medical Ethics Committee of Iwate Medical University (HG H25-2).

### GWAS genotyping and genotyping imputation

#### J-MICC

DNA was extracted from buffy coat with a BioRobot M48 Workstation (QIAGEN Group, Tokyo, Japan). The genotyping was conducted by the RIKEN institute (Yokohama, Japan) using an Illumina OmniExpressExome Array (Illumina, San Diego, CA, USA) for the 964,193 SNPs. We excluded 26 samples with inconsistent sex information between questionnaire and an estimate from genotype. The identity-by-descent method implemented in the PLINK 1.9 software (https://www.cog-genomics.org/plink2) found 388 close relationship pairs (pi-hat > 0.1875) and one sample of each pair was excluded. Principal component analysis (PCA) with a 1,000 Genomes reference panel (phase 3) (http://www.internationalgenome.org/category/phase-3/) detected 34 subjects whose estimated ancestries were outside of the Japanese population, who were excluded from the analyses. All the remaining 14,091 samples met a sample-wise genotype call rate criterion (>0.99). SNPs with a genotype call rate <0.98 and/or a Hardy-Weinberg equilibrium exact test *P* value <1 × 10^−6^, a low minor allele frequency (MAF) <0.01, or a departure from the allele frequency computed from the 1,000 Genomes Phase 3 EAS (East Asian) samples were excluded. Quality control filtering resulted in 14,091 individuals and 570,162 SNPs.

Thereafter, those who withdrew their research consent were excluded, resulting in 14,083 participants for analysis. Among them, data for SCr were available for 11,681 subjects, and 398 subjects who had their creatinine measured with the Jaffe method, 8 subjects with eGFR <15 mL/min/1.73 m^2^, 3 subjects with measurement errors, and 4 subjects with study withdrawal were excluded, leaving 11,268 subjects (who had their creatitine measured with enzyme method) for the final analyses.

Genotype Imputation was conducted using SHAPEIT Version 2 (https://mathgen.stats.ox.ac.uk/genetics_software/shapeit/shapeit.html#home) and IMPUTE2 (https://mathgen.stats.ox.ac.uk/impute/impute_v2.html) software based on the 1,000 Genomes Project Phase 3 (all ethnic groups). After the genotype imputation, variants with MAF <0.01 or info <0.4 were excluded.

#### JPHC

DNA samples from participants were extracted from the buffy coat of the peripheral white blood cells using FlexiGene DNA kits (QIAGEN Group) and genotyped using the HumanOmniExpressExome-8 v1.2, BeadChip or HumanOmniExpress-12 BeadChip arrays (Illumina) for JPHC1, and the HumanOmniExpressExome-8 v1.2, BeadChip array (Illumina) for JPHC2. The Genotyping was done in three laboratories, the Genetics Division, National Cancer Center Research Institute, the Department of Clinical Genomics, Fundamental Innovative Oncology Core, National Cancer Center Research Institute, and the RIKEN Center for Integrative Medical Sciences. As SNP quality control, SNPs with call rate <0.99, Hardy-Weinberg equilibrium *P*-value <1 × 10^−6^, minor allele frequency <0.01, or departure from the allele frequency computed from the 1,000 Genomes Phase 3 EAS (East Asian) were excluded. We conducted imputation for the remaining 482,391 and 581,625 SNPs in JPHC1 and 2, respectively, using SHAPEIT and IMPUTE2 with the 1,000 Genome Project Phase 3 (all ethnic groups) as a reference panel.

After genotyping, we applied standard quality control of GWAS (insufficient sample call rate, sex mismatches, related samples, non-Japanese ancestry), and then 5,331 and 2,473 participants remained for JPHC1 and 2, respectively. We also excluded participants who had missing or outlier data (eGFR <15 mL/min/1.73 m^2^) for SCr levels. Eventually, 4,502 and 2,234 participants were included in the analysis in JPHC1 and 2, respectively.

#### TMM

DNA extraction and genotyping were performed as described previously (PMID: 31130587). Briefly, the Japonica Array Version 2 (JPAv2) was utilized to genotype 53,599 subjects. Quality control was conducted on the genotype data using PLINK version 1.90b5.1 to exclude participants who met the following criteria: a low call rate (<0.99), non-Japanese ancestry, or one of a close-kin pair (pi-hat > 0.1875). Subsequently, variants with a low call rate (<0.99), low Hardy-Weinberg equilibrium exact test *P*-values (*P* < 1 × 10^−6^), low minor allele frequency (<0.01), insertions or deletions (indels), or exhibiting deviation from the allele frequency calculated from the 1,000 Genomes Phase 3 JPT samples, were excluded. Variants with missing or strand errors were also detected by SHAPEIT2 and then removed. This quality control resulted in 40,797 individuals and 551,426 SNPs that remained and applied for imputation.

Genotype Imputation was conducted using SHAPEIT Version 2 (https://mathgen.stats.ox.ac.uk/genetics_software/shapeit/shapeit.html#home) and IMPUTE2 (https://mathgen.stats.ox.ac.uk/impute/impute_v2.html) software based on the 1,000 Genomes Project cosmopolitan reference panel (phase 3). After the genotype imputation, variants with MAF <0.01 or info <0.4 were excluded. Thereafter, subjects with severe CKD (eGFR <15 mL/min/1.73 m^2^) were excluded. Finally, 40,744 subjects with data for SCr and eGFR were applied for the GWAS.

### GWAS of BioBank Japan (BBJ) project

To increase the sample sizes, we obtained the GWAS summary statistics of the BBJ from the data repository of NBDC Human Database (https://humandbs.biosciencedbc.jp/hum0014-v26#58qt).^12^ The BBJ project is a large hospital-based case-control study launched in 2003 and collected DNA and clinical information from about 20,000 participants who visited about 130 hospitals throughout Japan,^13^ of whom 142,097 subjects had data for SCr and 143,658 subjects had data for eGFR after subjects with severe CKD (eGFR <15 mL/min/1.73 m^2^) were excluded from the analyses. The GWAS genotyping and imputation procedures are as described elsewhere.^14^

### Phenotypes

SCr levels of the study participants were analyzed mainly using an enzymatic method after exclusion of 398 subjects of J-MICC Study who had their SCr levels measured with the Jaffe method. The eGFR of each participant was calculated based on SCr, age, and sex using the Japanese eGFR equation proposed by the Japanese Society of Nephrology: eGFR (mL/min/1.73 m^2^) = 194 × SCr (mg/dL)^−1.094^ × age^−0.287^ (× 0.739 if female), which is the calibrated version from the Chronic Kidney Disease Epidemiology Collaboration.^15^ In the association analyses, the SCr values were common log-transformed and adjusted for age, sex, and top ten principal components (PCs) in a linear regression model, and then the residuals were normalized by applying *Z*-score transformation. The eGFR values were adjusted for age, sex, and top ten PCs in a linear regression model, and then the residuals were normalized by applying rank-based inverse normal transformation.^12^

### Association analysis of SNPs with SCr and eGFR

The association of the normalized residuals of SCr and eGFR with SNP allele dose was tested by linear regression analysis. The regression coefficients and its standard errors estimated in linear regression analysis were used in the subsequent analysis. All the GWAS association analyses were conducted with SNPTEST ver. 2.5.2 (https://mathgen.stats.ox.ac.uk/genetics_software/snptest/old/snptest.html).

To identify studies with inflated GWAS significance, which can result from population stratification, we computed the intercept from the linkage disequilibrium (LD) score regression.^16^ Before the meta-analysis, all study-specific results in the association analysis were corrected by multiplying the standard error of the regression coefficient by the value of intercept from LD score regression.

### GWAS meta-analysis and statistical analyses

The association results for each SNP across the studies were combined with METAL^17^ software using the fixed-effects inverse-variance-weighted method. The meta-analysis included SNPs for which summary statistics were available from at least two studies with a total sample size of at least 20,000 individuals for the GWAS meta-analysis. To assess the inflation of the test statistics for the meta-analysis, we computed the genomic inflation factor, *λ*, and intercept from LD score regression.^16^

The regional plots were constructed using the LocusZoom software (http://genome.sph.umich.edu/wiki/LocusZoom_Standalone).

The novel loci were identified with reference to the previously reported loci posted in GWAS Catalogue (https://www.ebi.ac.uk/gwas/), PheWeb (https://pheweb.jp/) and CKDGen databases (https://ckdgen.imbi.uni-freiburg.de/) and SNPs within a region of 1 Mb at either side of the reported SNPs were excluded. A locus was defined as a ±500 kb region centered on a lead SNP (SNP with the lowest *P* value among loci). When any two such loci overlapped, they were merged into a single locus. We explored further independent association signals at each associated locus for SCr or eGFR. First, among SNPs that showed genome-wide significant associations, SNPs with low LD with the lead SNP of *r*^2^ < 0.1 in the 1,000 genome phase 3 EAS were selected as independent signal candidates at the same locus. These candidate and lead SNPs were then selected in a stepwise method to identify genome-wide significant SNPs as independent signals with the lead SNP. We performed the stepwise selection using GCTA-COJO^18^ with the option “--cojo-slct”. The summary statistics of the GWAS meta-analysis and LD estimated from imputed genotype data from 14,088 samples from the J-MICC Study were used for the analyses. The genome-wide significance levels were set at *P* < 5 × 10^−8^ in all the analyses.

### Functional annotation

To prioritize the associated SNPs of the identified loci, we adopted a series of bioinformatic approaches to collate functional annotations. We first used ANNOVAR^19^ to obtain an aggregate set of functional annotations—including gene locations and impacts of amino acid substitutions based on prediction tools, such as SIFT, PolyPhen-2, and CADD—for SNPs with genome-wide or suggestive significance for kidney functions around *CD36* gene.

## RESULTS

### Characteristics of the study participants

The characteristics of the study subjects analyzed are shown in Table 1. A total of 202,406 participants were included in the analyses for SCr and 200,845 participants for eGFR. The mean value of SCr in BBJ, a hospital-based case-control study, was 0.77 mg/dL, which was slightly higher than those of other cohorts (0.72, 0.70, 0.72 and 0.69 mg/dL in J-MICC, JPHC1, JPHC2 and ToMMo, respectively), while the mean values of eGFR were 73.9 mL/min/1.73 m^2^ in BBJ, and 78.7, 76.7, 76.8 and 78.8 mL/min/1.73 m^2^ in J-MICC, JPHC1, JPHC2, and ToMMo, respectively.

**Table 1.** Study characteristics

(Cohort)	(Study characteristics)							

	SCr				eGFR			
	
	*n*	male, *n* (%)	Age, years, mean (SD)	SCr, mg/dL, mean (SD)	*n*	Male, *n* (%)	Age, years, mean (SD)	eGFR, mL/min/1.73 m^2^, mean (SD)
J-MICC	11,268	5,148 (45.7%)	54.9 (9.3)	0.72 (0.17)	11,268	5,148 (45.7%)	54.9 (9.3)	78.8 (14.9)

JPHC1	4,502	1,356 (30.1%)	62.7 (7.1)	0.70 (0.17)	4,502	1,356 (30.1%)	62.7 (7.1)	76.7 (30.9)
JPHC2	2,234	871 (39.0%)	62.2 (7.2)	0.72 (0.20)	2,234	871 (39.0%)	62.2 (7.2)	76.8 (30.8)

TMM	40,744	15,766 (38.7%)	60.67 (10.97)	0.69 (0.17)	40,744	15,766 (38.7%)	60.67 (10.97)	78.8 (16.0)

BBJ	142,097	- (54.8%)^a^	62.9 (13.2)	0.77 (0.22)	143,658	- (54.9%)^a^	62.9 (13.2)	73.9 (15.4)

BBJ, BioBank Japan; eGFR, estimated glomerular filtration rate; J-MICC, Japan Multi-Institutional Collaborative Cohort Study; JPHC, Japan Public Health Center-based Prospective Study; TMM, Tohoku Medical Megabank Community-Based Cohort Study; SCr, serum creatinine; SD, standard deviation.

^a^The numbers of male subjects in BBJ are unknown.

### Novel genetic loci identified in the GWAS meta-analysis

A total of about 200,000 individuals were included in these GWAS meta-analyses of Japanese studies. The genomic inflation factor, λ for meta-analysis of SCr and eGFR were 1.31 and 1.31, respectively. The LD score regression intercepts of the summary statistics for meta-analyses of SCr and eGFR were 1.05 and 1.06, respectively (eTable 1). These values revealed no evidence of genomic inflation for meta-analyses.

**Figure 1.**
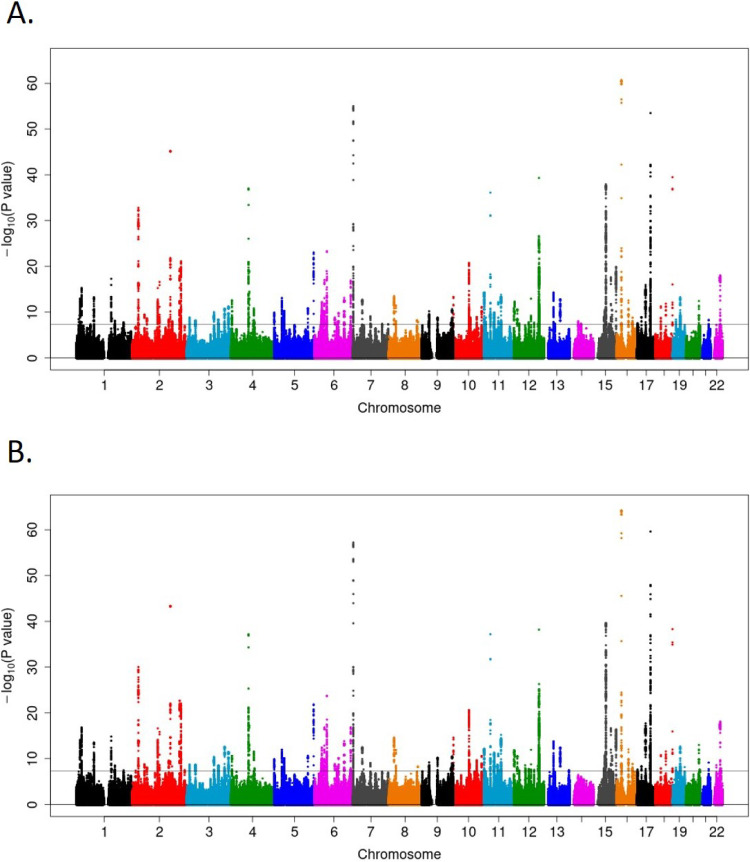
Manhattan plots for the GWAS meta-analyses of kidney functions in Japanese (**A**: SCr, **B**: eGFR). eGFR, estimated glomerular filtration rate; SCr, serum creatinine.

**Figure 2.**
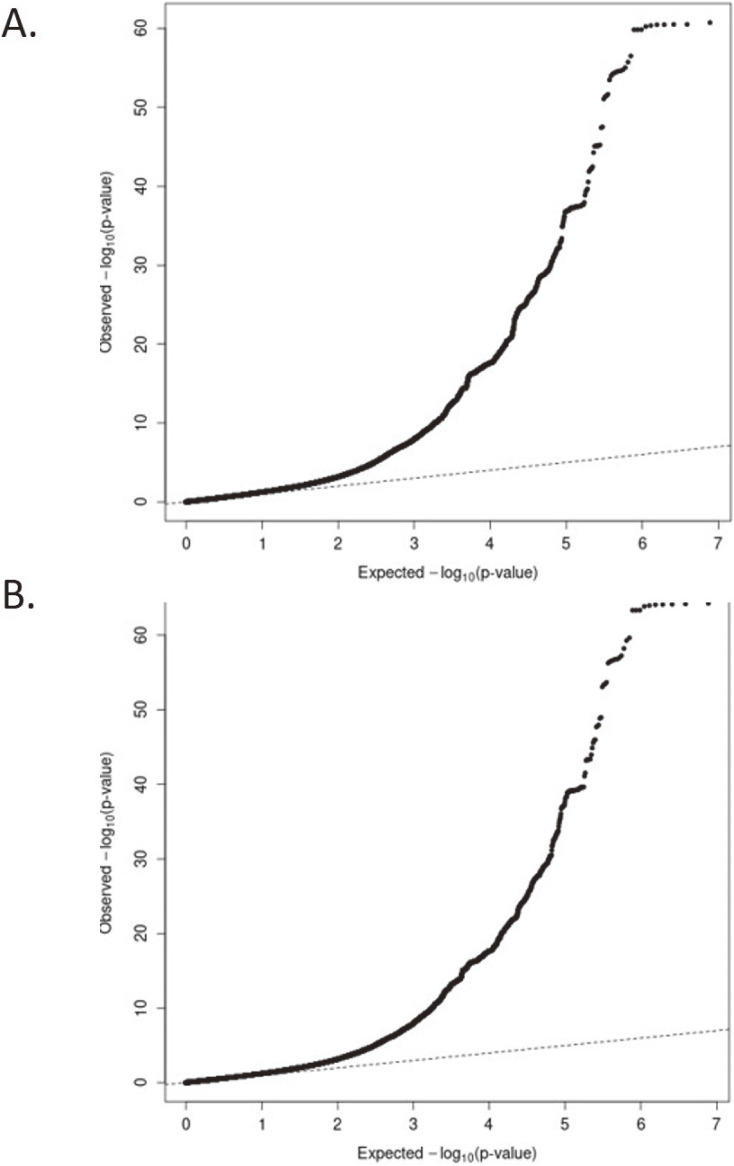
Q-Q plots for the GWAS meta-analyses of kidney functions in Japanese (**A**: SCr, **B**: eGFR). eGFR, estimated glomerular filtration rate; SCr, serum creatinine.

**Figure 3.**
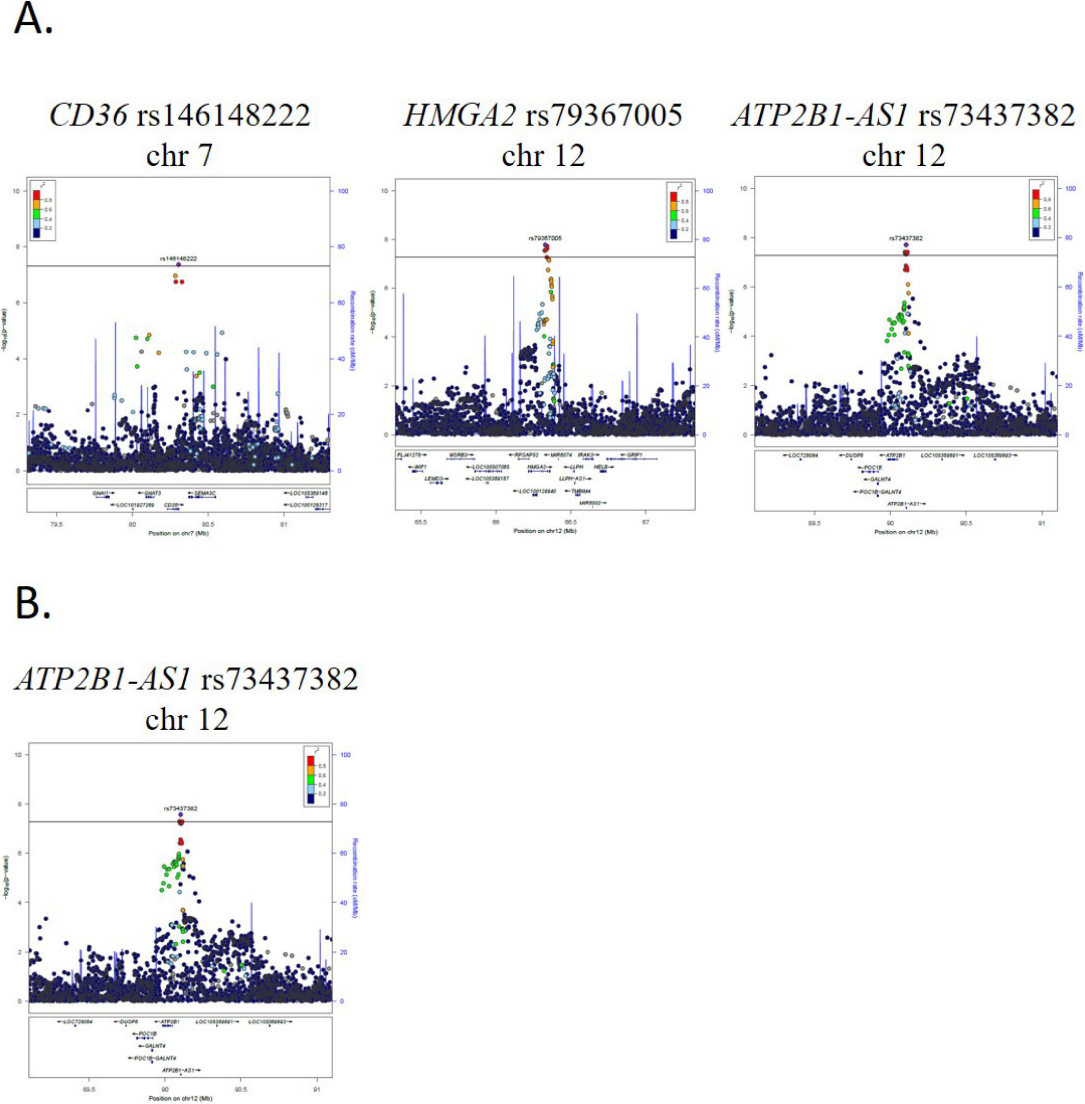
Regional plots for the novel loci of kidney functions in Japanese (**A**: SCr, **B**: eGFR). eGFR, estimated glomerular filtration rate; SCr, serum creatinine.

In the present GWAS meta-analysis of representative large genome cohort studies in Japan, we identified 110 loci with 169 variants significantly associated with eGFR (on chromosomes 1–13 and 15–22; *P* < 5 × 10^−8^), whereas we also identified 112 loci with 176 variants significantly associated with SCr (on chromosomes 1–22; *P* < 5 × 10^−8^), of which one novel locus (more than 1 Mb distant from known loci) with one variant (*CD36* [*Cluster of Differentiation 36*] rs146148222 on chromosome 7) for SCr, together with two loci with two variants for SCr (*HMGA2* [*High mobility group AT-HOOK 2*] rs79367005 and *ATP2B1-AS1* [*ATP2B1 Antisense RNA1*] rs73437382 on chromosome 12) and one locus with one variant for eGFR (*CD36* rs146148222 on chromosome 7) which were more than 100 kb distant from known loci, were considered as original findings (Table 2). The Manhattan Plots for these analyses are shown in Figure 1. The Q-Q plots for the *P*-values in each of these analyses are presented in Figure 2. Regional plots for the novel loci are provided in Figure 3. The previously reported loci and the independent signals are summarized in eTable 2, eTable 3, eTable 4, and eTable 5.

**Table 2.** Novel genetic variants significantly associated with renal functions (SCr and eGFR) (*P* < 5 × 10^−8^) in the GWAS meta-analyses of kidney functions in Japanese

Trait	rsID	Chr	Position	Gene	Function	Effect allele	Other allele	*N*	EAF	Beta	SE	*P*	I^2^	HetPVal
**SCr**														
Novel^a^														
	rs146148222	7	80304855	*CD36*	intronic	T	G	200845	0.952	0.045	0.008	4.36E-08	70.1	0.010
Semi-novel^b^														
	rs79367005	12	66328866	*HMGA2*	intronic	T	C	200845	0.112	−0.029	0.005	1.64E-08	54.3	0.067
	rs73437382	12	90102945	*ATP2B1-AS1*	ncRNA_exonic	C	G	200845	0.728	0.021	0.004	1.94E-08	0	0.439
**eGFR**														
Semi-novel^b^														
	rs73437382	12	90102945	*ATP2B1-AS1*	ncRNA_exonic	C	G	202406	0.728	−0.021	0.004	2.67E-08	0	0.552

Beta, regression coefficient; Chr, chromosome; EAF, effect allele frequency; eGFR, estimated glomerular filtration rate; GWAS, genome-wide association study; HetP, *P*-value for heterogeneity; SCr, serum creatinine; SE, standard error.

^a^genetic loci more than 1 Mb distant from known loci.

^b^genetic loci more than 100 kb distant from known loci.

## DISCUSSION

To the best of our knowledge, the present study is the first GWAS meta-analysis of representative large genome cohort studies sized ≥100,000 study participants in Japan, which may provide beneficial information for the effective prevention of kidney diseases based on genetic information in Japanese. The present study revealed one novel genetic loci (>1 Mb away from known loci) associated with SCr, the genetic locus of CD36, and two semi-novel genetic loci (>100 kb away from known loci) associated with eGFR and/or SCr, which included *ATP2B1-AS1* and *HMGA2*. Among the newly found loci, *ATP2B1-AS1* is an RNA gene affiliated with the lncRNA class. Diseases associated are Glioma Susceptibility 1 and Epidural Spinal Canal Neoplasm,^19^^–^^24^ *HMGA2* contains structural DNA-binding domains and may act as a transcriptional regulator.

*CD36* encodes a fourth major glycoprotein on the platelet surface and works as a cell adhesion molecule which binds to collagen, thrombospondin (TSP) and oxidized low density lipoprotein (LDL) cholesterol. Mutation of *CD36* leads to platelet glycoprotein deficiency.

Accumulating evidence has shown the critical roles of CD36 molecule in the development of CKD in humans. CD36 gene is located on chromosome 7q11.2; mutations in CD36 gene are linked to abnormal plasma fatty acid and triglycerides, and metabolic disorders involving insulin resistance. It is shown that kidney extracts fatty acids from circulation and fatty acid oxidation (FAO) accounts for more than 50% of kidney oxygen consumption. CD36 is responsible for oxidized LDL uptake in macrophages, and in mice with kidney injury oxidized LDL deposition is observed in kidney tubules which is correlated with fibrosis.^25^ Advanced oxidation protein products (AOPPs) are important kidney pathogenic mediators in the progression of CKD, and CD36 binding of AOPPs is shown to activate the RNA system in proximal tubules.^26^ Interaction of TSP1 with CD36 is involved in cellular fibrosis.^27^ Advanced glycation end-products (AGEs) have important roles in the development of diabetic nephropathy, and AGE-LDL has been shown to produce pro-inflammatory cytokines by interaction with CD36.^28^ CD36 has also been reported to have a role in serum amyloid A protein (SAA) induced inflammation in human kidney cell line.^29^ A reduction in FAO in CKD has an important role in kidney fibrosis by disrupting the balance between fatty acid synthesis, intake, and consumption. Mitochondrial transfer of fatty acids is the rate limiting step in FAO, and CD36 is considered to play important roles in FAO through its function in the mitochondrial transfer of fatty acids.^30^ Given those crucial roles of CD36 in the development of kidney injury, the significant association of CD36 SNP with the kidney function in the present Japanese GWAS sounds biologically plausible. Meanwhile, although functional significance of the CD36 rs146148222 SNP found in the present study remains yet unknown because this SNP is not included in the GTEx expression database, a number of functional SNPs in CD36 gene are in LD with the CD36 SNP found in the present GWAS, suggesting the etiological involvement of this SNP in kidney functions of Japanese subjects. Additionally, we first searched on ANNOVAR^31^ to investigate the functional annotations of the variants of *CD36* gene including the *CD36* rs146148222 SNP found in this study, and then searched on Nephroseq^32^ (a database of omics data related to kidney function based on existing studies) to see if there is any difference in CD36 expressions in patients with CKD and without CKD. As a result of our analyses, it was predicted that rs75326924 SNP, an East Asian-specific SNP which is in LD with rs146148222 found in the present study (*D*′ = 1.0, *r*^2^ = 0.8955), may also be a damaging causal variant (eTable 6), and it might affect CD36 protein functions through the alteration of protein structure or protein amount by way of the LD status with some regulatory SNPs in the 5′UTR region of *CD36* gene such as rs2366855 (*D*′ = 1.0, *r*^2^ = 0.0128) or rs1049654 (*D*′ = 1.0, *r*^2^ = 0.0128), and resultant SCr as well, considering the significant reduction in the expressions of CD36 in CKD kidneys compared with those in normal kidneys based on Nephroseq (eFigure 1).^33^ Our in-silico analyses with Nephroseq might suggest that the compromised CD36 lipid scavenger function due to the *CD36* SNPs might lead to elevated risk of CKD. Further investigations to clarify the roles of this CD36 in the prevention and treatment of CKD are warranted for the possible development of personalized prevention and medical treatment of CKD in the near future.

Although the present study found several novel loci associated with kidney functions in Japanese, importance of the replicability of previously reported loci is recently reemphasized under the cautious eyes of the researchers intending to avoid underpowered studies.^34^^,^^35^ As expected, the present GWAS meta-analyses of representative Japanese large genome cohorts successfully replicated the reported loci with considerably high reproducibility. For example, *PDILT* rs35208507 on chr16, *BCAS3* rs9895661 on chr17, *UNCX* rs10277115 on chr7 and *NFATC1* rs549752 on chr18 in association with eGFR were replicated with strikingly high significance with *P*-values of 5.7 × 10^−65^, 2.4 × 10^−60^, 6.0 × 10^−58^ and 4.9 × 10^−39^, and *PDILT* rs35208507 on chr16, *UNCX* rs10277115 on chr7, *BCAS3* rs9895661 on chr17 *LRP2* rs3770636 on chr2 and *NFATC1* rs549752 on chr18 in association with SCr were replicated with *P*-values of 1.9 × 10^−61^, 9.4 × 10^−56^, 3.5 × 10^−54^, 5.8 × 10^−46^ and 3.2 × 10^−40^, respectively.

The present study has some strengths and limitations to be mentioned. To our knowledge, this is one of the largest GWAS meta-analyses ever that investigated the genetic factors associated with kidney function traits in Japanese, leveraging the GWAS summary statistics of three representative large genomic cohorts in Japan, together with those from the open data repository of BBJ. Especially, the present GWAS meta-analysis consisted of population-based cohort participants, which should be advantageous compared to the hospital-based case-control studies because the participants were considered to be free from the influence of pre-existing diseases. Investigations of population-specific loci for ancestries/ethnicities would be considered meaningful, which may lead to the possible clarifications of effective ways of disease prevention for each population.^36^ The present study results suggested that almost all loci are common across different populations, whereas the lead SNPs are partially different between populations.

With regard to the limitations, whereas the present study clarified novel genetic loci associated with eGFR and SCr, the mechanisms in which those genetic loci exert their effects remain largely unknown. The different finding between novel loci of eGFR and those of SCr observed might be considered a result of the influence of adjustments for age and gender in the Japanese eGFR estimation formula.^15^ The present GWAS of kidney function traits is limited to Japanese ancestry; larger GWAS with multiple ancestries may enable the comparisons of the reasonable SNPs for CKD risk characteristic of each population. Meanwhile, the results of GWAS for estimated GFR from serum Cr are often affected by creatinine metabolism, so future validation studies considering other estimation method of GFR, such as cystatin C measurements, should be warranted to confirm the present study results. With regard to the technical aspects, batch effects are not assumed because all the GWAS genotyping was conducted with the same SNP chip in the same institute (Illumina OmniExpressExome array & RIKEN). More specifically and precisely, all the cohorts except for JPHC1 used Illumina OmniExpressExome array, and JPHC1 is OmniExpress-based, but OmniExpressExome are mixed. However, in JPHC, the population to be typed is selected by random sampling from the cohort, regardless of cases and controls, so batch effects are unlikely to occur. Further epidemiological studies with larger samples in Japanese as well as East Asians and biological studies for their clarification are warranted.

In conclusion, the present GWAS meta-analysis of largest genome cohort studies in Japanese provided novel genomic loci associated with kidney function in Japanese, which may contribute to the future interplay of epidemiologists, biologists, and medical practitioners for the possible development of personalized prevention of kidney diseases based on genomic information in the near future.

## SUPPLEMENTARY MATERIAL

eTable 1. Results of the LD score regression (LDSC)

eTable 2. Previously reported variants significantly associated with eGFR (*P* < 5 × 10^−8^) in the GWAS meta-analyses of kidney functions in Japanese

eTable 3. Previously reported variants significantly associated with SCr (*P* < 5 × 10^−8^) in the GWAS meta-analyses of kidney functions in Japanese

eTable 4. Independent signals significantly associated with eGFR (*P* < 5 × 10^−8^) in the GWAS meta-analyses of kidney functions in Japanese

eTable 5. Independent signals significantly associated with SCr (*P* < 5 × 10^−8^) in the GWAS meta-analyses of kidney functions in Japanese

eTable 6. Allele frequency, effect size, and functional annotation for four SNPs with genome-wide or suggestive significance around *CD36* gene

eFigure 1. Expression of CD36 in the kidneys of healthy subjects and CKD patients. CKD, chronic kidney disease.
