# Supplementary material for: GWAS Meta-analysis of Kidney Function Traits in Japanese Populations
Source: J Epidemiol. 2024 Nov 5;34(11):526–34. doi: 10.2188/jea.JE20230281 (PMC11464852; doi:10.2188/jea.JE20230281)
Supplement: Supplementary file 1 [file je-34-526-s001.pdf]

eTable 1. Results of the LD score regression (LDSC)

**Trait**	**SNP (*n*)**	h^2_g	**SE**	λ_{GC}	**Mean** χ^2	**Intercept**	**SE**	**Ratio**	**SE**
SCr	1,012,083	0.1149	0.0076	1.3101	1.5545	1.0527	0.0124	0.0951	0.0223
eGFR	1,012,083	0.1132	0.0075	1.3101	1.5535	1.0553	0.0121	0.0998	0.0219

eGFR, estimated glomerular filtration rate; LD, linkage disequilibrium; LDSC, LD score regression; SCr, serum creatinine; SE, standard error; SNP, single-nucleotide polymorphism.

eTable 2. Previously reported variants significantly associated with eGFR ($P<5\times10^{-8}$) in the GWAS meta-analyses of kidney functions in Japanese

rsID	Chr	Position	Gene	Function	Effect allele	Other allele	N	EAF	Beta	SE	*P*	I2	HetPVal	Reported in JP/EAS	Reported in CEU/TE	PMID
rs848301	1	16315262	*ZBTB17, SRARP*	intergenic	C	G	202406	0.332	-0.029	0.004	7.60E-15	0	0.410	Y	Y[a]	20604766; 34594039; 29403010
rs12563200	1	23680630	*HNRNPR, ZNF436*	intergenic	T	C	202406	0.204	0.035	0.004	1.45E-17	63.8	0.026	Y	Y	31015462
rs278853	1	78130243	*ZZZ3*	intronic	T	C	202406	0.398	0.026	0.003	2.64E-14	69.1	0.012	Y[a]	Y[a]	36329257
rs2990246	1	155197602	*GBAP1*	upstream	C	G	202406	0.843	0.039	0.005	1.38E-15	76.9	0.002	Y	Y[a]	29403010; 31015462
rs34720381	1	171455322	*PRRC2C*	intronic	T	C	202406	0.069	-0.039	0.007	3.42E-09	0	0.630	Y	Y	29403010; 31152163
rs3111244	1	211395345	*KCNH1, RCOR3*	intergenic	A	G	202406	0.580	-0.019	0.003	3.55E-08	0	0.887	Y[a]	Y[a]	34594039
rs807624	2	15782471	*DDX1, LINC01804*	intergenic	T	G	202406	0.795	0.022	0.004	4.04E-08	0	0.698	Y	Y	31152163; 34272381

rs1260326	2	27730940	*GCKR*	exonic	T	C	202406	0.558	0.038	0.003	9.56E-31	59.3	0.044	Y	Y	27588450;34594039
rs1527649	2	54581356	*C2orf73*	intronic	T	C	202406	0.743	0.023	0.004	1.62E-09	61.6	0.034	Y	Y	30604766
rs2118304	2	65681937	*SPRED2, MIR4778*	intergenic	A	G	202406	0.856	0.029	0.005	8.00E-09	29.2	0.227	Y[a]	Y[a]	35710981
rs11123169	2	113967075	*PSD4*	downstream	T	C	202406	0.731	0.031	0.004	2.29E-17	0	0.826	Y	Y	29403010;34272381
rs7596689	2	122039095	*TFCP2L1*	intronic	A	C	202406	0.946	0.060	0.007	1.33E-16	0	0.732	Y	Y[a]	27588450;34594039
rs12473366	2	163075264	*FAP*	intronic	A	T	202406	0.871	-0.032	0.005	2.58E-10	0	0.952	Y[a]	Y[a]	34594039
rs3770636	2	170202833	*LRP2*	intronic	T	G	202406	0.806	-0.059	0.004	4.23E-44	81.8	0.000	Y	Y	31015462
rs1005932	2	177331710	*MTX2, MIR1246*	intergenic	A	G	202406	0.355	0.020	0.003	4.72E-09	0	0.719	Y	Y[a]	33462484
rs55932961	2	211549856	*CPS1, ERBB4*	intergenic	A	G	202406	0.156	-0.046	0.005	2.08E-23	56.9	0.054	Y	Y	29403010;34594039
rs7598172	2	217677999	*IGFBP5, TNP1*	intergenic	T	G	202406	0.511	-0.032	0.003	8.88E-23	83.1	0.000	Y	Y[a]	29403010;

																28452372
rs283482	2	233646920	*GIGYF2*	intronic	A	T	202406	0.579	-0.019	0.003	3.02E-08	28.9	0.229	Y[a]	Y[a]	34594039
rs182545	3	12192525	*SYN2*	intronic	C	G	202406	0.648	0.021	0.003	1.94E-09	0	0.785	Y[a]	Y[a]	34594039; 31152163
rs13095391	3	38447232	*XYLB*	intronic	A	C	202406	0.348	-0.021	0.004	1.88E-09	0	0.773	Y[a]	Y[a]	34272381
rs2332050	3	121635818	*SLC15A2*	intronic	C	G	202406	0.268	0.025	0.004	4.39E-11	30.6	0.217	Y	Y	29403010; 34272381
rs6764769	3	141100280	*ZBTB38*	UTR5	A	G	202406	0.642	0.019	0.003	1.26E-08	0	0.441	Y[a]	Y[a]	30604766
rs13065446	3	141737842	*TFDP2*	intronic	T	C	202406	0.709	-0.022	0.004	2.89E-09	0	0.877	Y	Y[a]	31152163; 30604766
rs16853722	3	169150632	*MECOM*	intronic	T	C	202406	0.722	0.027	0.004	2.23E-13	4.9	0.379	Y	Y[a]	29403010; 31152163
rs11927941	3	186433046	*HRG, KNG1*	intergenic	A	G	202406	0.702	0.025	0.004	3.25E-12	36.6	0.177	Y	Y	30604766
rs13108218	4	3443931	*HGFAC*	intronic	A	G	202406	0.546	0.022	0.003	2.52E-11	15.3	0.317	Y	Y	30604766
rs2279252	4	5800494	*EVC*	exonic	A	G	202406	0.097	0.036	0.006	9.90E-11	28.7	0.230	Y	Y	35710981

rs10025351	4	77394095	*SHROOM3*	intronic	T	C	202406	0.216	-0.051	0.004	7.00E-38	0	0.831	Y	Y[a]	29403010; 31152163
rs16998073	4	81184341	*PRDM8, FGF5*	intergenic	A	T	202406	0.700	-0.031	0.004	5.65E-18	0	0.438	Y	Y	36329257; 31451708
rs3765637	4	101114121	*LOC101929353*	ncRNA_splicing	A	T	202406	0.537	0.023	0.003	2.63E-12	0	0.843	Y[a]	Y[a]	29403010; 35710981
rs223413	4	103732866	*UBE2D3*	intronic	T	C	202406	0.357	0.019	0.003	1.26E-08	9	0.355	Y	Y	30604766
rs688334	5	682908	*TPPP*	intronic	T	C	202406	0.411	-0.024	0.004	1.31E-10	29.4	0.226	Y[a]	Y[a]	31152163
rs7714709	5	34510387	*NONE, RAI14*	intergenic	A	G	202406	0.741	0.028	0.004	9.73E-13	0	0.765	Y	Y[a]	29403010
rs72753700	5	44117677	*NNT, FGF10*	intergenic	A	G	202406	0.246	-0.025	0.004	8.17E-11	0	0.476	Y[a]	Y[a]	31152163
rs118169288	5	151157909	*G3BP1*	intronic	A	G	202406	0.086	0.042	0.006	2.32E-11	0	0.911	Y[a]	Y[a]	34594039
rs3812036	5	176813404	*SLC34A1*	intronic	T	C	202406	0.251	-0.037	0.004	1.35E-22	69.6	0.011	Y	Y	29403010; 28452372
rs9368805	6	34225075	*SMIM29, NUDT3*	intergenic	T	C	202406	0.218	-0.026	0.004	4.98E-10	53	0.074	Y	Y[a]	29403010; 35213538

rs35985639	6	41673580	*TFEB*	intronic	A	G	202406	0.271	0.024	0.004	1.67E-10	0	0.664	Y	Y[a]	29403010;33462484
rs881858	6	43806609	*VEGFA, LINC02537*	intergenic	A	G	202406	0.878	-0.042	0.005	1.49E-17	80.6	0.000	Y	Y	27588450
rs2143081	6	50782834	*TFAP2D, TFAP2B*	intergenic	A	G	202406	0.576	0.019	0.003	3.62E-09	0	0.642	Y	Y	35710981
rs4715517	6	54973761	*HCRTR2*	intronic	A	C	58748	0.085	-0.117	0.012	1.75E-24	25.9	0.256	Y	Y	34594039
rs6907843	6	90109374	*RRAGD*	intronic	T	C	202406	0.084	-0.036	0.006	1.47E-09	0	0.439	Y	Y	34594039
rs6570797	6	101044623	*ASCC3*	intronic	A	G	202406	0.637	-0.020	0.004	5.29E-09	20.2	0.286	Y[a]	Y[a]	34594039
rs7766720	6	107172979	*LINC02532*	ncRNA_intronic	T	C	202406	0.819	0.030	0.005	7.28E-11	59.6	0.042	Y	Y	31152163
rs2781656	6	131882571	*AKAP7, ARG1*	intergenic	T	C	202406	0.320	-0.027	0.004	1.39E-14	7.2	0.366	Y	Y[a]	29403010
rs62430664	6	133842133	*TARID*	ncRNA_intronic	T	C	58748	0.907	-0.057	0.010	3.13E-08	0	0.576	Y[a]	Y[a]	33462484;35710981
rs533452	6	160697762	*SLC22A2, SLC22A3*	intergenic	A	G	197904	0.108	0.045	0.005	1.37E-17	43.8	0.149	Y[a]	Y[a]	29403010;28452372

rs10277115	7	1285195	*UNCX, MICALL2*	intergenic	A	T	202406	0.683	0.057	0.004	5.98E-58	34.9	0.189	Y	Y	22797727; 26831199
rs78503047	7	41068572	*LINC01450, LINC01449*	intergenic	T	C	202406	0.117	-0.037	0.005	2.84E-13	33.4	0.199	Y	Y	31152163
rs856557	7	46758306	*LOC730338, TNS3*	intergenic	A	T	58748	0.762	-0.042	0.007	2.24E-08	9.8	0.344	Y[a]	Y[a]	35710981
rs10263369	7	77406404	*RSBN1L*	intronic	T	C	202406	0.233	-0.024	0.004	9.65E-10	0.5	0.403	Y[a]	Y[a]	26831199; 35710981
rs9314272	8	23722263	*STC1, ADAM28*	intergenic	A	G	202406	0.181	-0.034	0.004	2.29E-15	0	0.903	Y[a]	Y[a]	35710981
rs4422737	8	32399849	*NRG1*	intronic	A	G	202406	0.832	0.031	0.004	1.09E-12	44.4	0.126	Y	Y	34594039
rs75834729	8	130371638	*CCDC26*	ncRNA_intronic	T	C	202406	0.287	0.021	0.004	4.90E-09	55.1	0.063	Y	Y[a]	29403010
rs62555928	9	33882692	*UBE2R2*	intronic	T	C	202406	0.510	0.020	0.003	6.45E-10	0	0.565	Y[a]	Y[a]	34272381
rs7048566	9	71507938	*PIP5K1B*	intronic	A	G	202406	0.291	-0.023	0.004	6.51E-11	51.1	0.085	Y[a]	Y[a]	28452372; 27588450
rs1043060	9	130705120	*FAM102A*	UTR3	T	C	202406	0.285	0.021	0.004	1.02E-08	40.6	0.151	Y	Y	34594039
rs9411378	9	136145425	*ABO*	intronic	A	C	202406	0.260	0.026	0.004	3.16E-11	14.2	0.324	Y[a]	Y[a]	34594039

rs75048142	9	140162664	*NELFB*	intronic	T	G	202406	0.092	-0.037	0.006	1.83E-09	34.3	0.193	Y[a]	Y[a]	35710981
rs80282103	10	899071	*LARP4B*	intronic	A	T	202406	0.923	0.050	0.006	2.35E-15	68.3	0.013	Y	Y	31152163; 28452372
rs7475348	10	69965177	*MYPN*	intronic	T	C	202406	0.321	0.034	0.004	2.30E-21	0	0.863	Y	Y	29403010; 31152163
rs6892	10	104575870	*WBP1L*	UTR3	A	G	202406	0.733	-0.023	0.004	2.27E-10	24.4	0.259	Y[a]	Y[a]	30604766
rs10901809	10	126410804	*FAM53B*	intronic	T	C	202406	0.289	0.024	0.004	2.46E-11	39.9	0.155	Y[a]	Y[a]	30604766
rs10840341	11	2116493	*H19, IGF2*	intergenic	A	T	202406	0.542	-0.024	0.003	6.53E-13	0	0.645	Y	Y	29403010; 35710981
rs963837	11	30749090	*MPPED2, DCDC1*	intergenic	T	C	202406	0.651	-0.047	0.004	6.30E-38	27.7	0.237	Y	Y	29403010; 30604766
rs61897431	11	47427667	*MIR4487, SLC39A13*	intergenic	T	C	202406	0.742	0.026	0.004	4.53E-12	9.8	0.350	Y	Y	31152163
rs174529	11	61543961	*MYRF*	intronic	T	C	202406	0.606	-0.021	0.003	2.04E-09	0	0.598	Y	Y	34594039
rs7123489	11	65524252	*RNASEH2C, AP5B1*	intergenic	A	C	202406	0.161	-0.	0.005	8.44E-12	0	0.787	Y	Y	31015462

									031							
rs11263642	11	69147825	*MYEOV, LOC102724265*	intergenic	A	G	202406	0.983	0.069	0.013	4.37E-08	0	0.435	Y[a]	Y[a]	31451708
rs7940841	11	78079084	*GAB2*	intronic	A	G	202406	0.599	-0.030	0.004	6.01E-16	40	0.155	Y	Y	29403010; 31152163
rs11062070	12	343632	*SLC6A13*	intronic	T	C	202406	0.412	-0.024	0.003	1.27E-12	0	0.457	Y	Y[a]	29403010
rs632887	12	3392351	*TSPAN9*	UTR3	A	G	202406	0.709	0.026	0.004	1.54E-12	0	0.490	Y	Y	30604766
rs7302787	12	15344066	*RERG*	intronic	T	G	202406	0.358	0.023	0.003	2.87E-11	0	0.460	Y	Y	31451708
rs10771021	12	23875372	*SOX5*	intronic	T	C	202406	0.188	-0.023	0.004	3.82E-08	25.2	0.254	Y	Y	35710981
rs836968	12	50267335	*FAIM2*	intronic	T	C	202406	0.546	0.020	0.003	2.88E-09	0	0.785	Y	Y	34594039
rs7962469	12	52348259	*ACVR1B*	intronic	A	G	202406	0.393	0.019	0.003	2.02E-08	0	0.413	Y[a]	Y[a]	35710981
rs58569716	12	66341873	*HMGA2*	intronic	T	C	202406	0.129	0.027	0.005	1.71E-08	49.5	0.094	Y[a]	Y[a]	35710981
rs1275609	12	76271183	*KRR1, PHLDA1*	intergenic	A	G	197904	0.597	0.025	0.004	1.08E-12	0	0.631	Y	Y	29403010; 30604766
rs79105258	12	111718231	*CUX2*	intronic	A	C	202406	0.244	-0.	0.004	6.30E-39	68.6	0.013	Y	Y	29403010;

									053							30604766
rs12430974	13	42737100	*DGKH*	intronic	A	G	202406	0.569	-0.026	0.003	1.58E-14	0	0.657	Y	Y[a]	29403010
rs626277	13	72347696	*DACH1*	intronic	A	C	202406	0.152	-0.033	0.005	3.34E-13	0	0.539	Y	Y	29403010
rs4555010	13	111049838	*COL4A2*	intronic	T	C	202406	0.686	0.019	0.004	3.43E-08	0	0.496	Y[a]	Y[a]	34594039
rs7168414	15	45639612	*LOC101928414, GATM*	intergenic	T	C	202406	0.842	-0.028	0.005	1.11E-08	25.7	0.250	Y[a]	Y[a]	31152163
rs35073878	15	51080002	*SPPL2A, AP4E1*	intergenic	T	C	202406	0.203	0.028	0.004	1.03E-11	61.4	0.035	Y	N	34594039
rs10518733	15	53940307	*WDR72*	intronic	A	C	202406	0.607	-0.044	0.003	2.30E-40	27	0.241	Y	Y[a]	20139978
rs12148134	15	67592098	*IQCH*	intronic	C	G	202406	0.417	-0.021	0.004	1.66E-08	3.8	0.385	Y[a]	Y[a]	31015462
rs11636251	15	76239020	*NRG4*	intronic	T	C	202406	0.501	-0.030	0.004	2.09E-17	12	0.337	Y	Y	30604766
rs8027238	15	81164723	*CEMIP*	intronic	T	C	202406	0.749	-0.026	0.004	2.34E-11	5.8	0.374	Y[a]	Y[a]	29403010
rs148840914	15	99306048	*IGF1R*	intronic	A	G	202406	0.266	-0.031	0.004	3.70E-17	45.3	0.120	Y[a]	Y[a]	29403010; 33462484

rs35208507	16	20388929	*PDILT*	intronic	A	G	202406	0.779	-0.067	0.004	5.70E-65	42.6	0.138	Y	Y[a]	34594039
rs9935770	16	21091291	*DNAH3*	intronic	T	C	202406	0.480	-0.022	0.003	2.70E-11	0	0.644	Y[a]	Y[a]	35710981
rs12935539	16	51754991	*SALL1, LINC01571*	intergenic	T	C	202406	0.685	0.025	0.004	8.65E-13	46.3	0.114	Y	Y	29403010; 30604766
rs12444699	16	69823443	*WWP2*	intronic	A	G	202406	0.870	-0.033	0.005	1.47E-09	63.4	0.027	Y[a]	Y[a]	31152163
rs12952051	17	1982790	*SMG6*	intronic	T	C	202406	0.232	-0.022	0.004	2.32E-08	0	0.802	Y	Y[a]	35710981
rs7222869	17	19417761	*RNF112, SLC47A1*	intergenic	A	G	202406	0.936	0.041	0.007	4.65E-09	14.1	0.324	Y	Y[a]	33462484; 29403010
rs7212715	17	37607904	*MED1*	upstream	T	C	202406	0.767	-0.038	0.004	1.78E-18	0	0.993	Y	Y[a]	29403010; 34272381
rs9895661	17	59456589	*BCAS3*	intronic	T	C	202406	0.469	0.059	0.004	2.41E-60	87.4	0.000	Y	Y	28452372; 27588450
rs16942751	18	24393213	*PCAT18, AQP4*	intergenic	A	C	202406	0.298	-0.024	0.004	1.72E-11	0	0.914	Y	Y	29403010; 30604766

rs2337106	18	46460903	*SMAD7*	intronic	C	G	202406	0.471	-0.023	0.003	2.55E-12	18	0.300	Y	Y	30604766
rs549752	18	77158225	*NFATC1*	intronic	A	G	202406	0.318	-0.047	0.004	4.91E-39	22.7	0.270	Y	Y[a]	28452372
rs2241359	19	14586245	*PTGER1*	upstream	A	G	202406	0.689	-0.020	0.004	9.27E-09	0	0.638	Y[a]	Y[a]	35710981
rs8101881	19	33364628	*SLC7A9, CEP89*	intergenic	T	C	202406	0.337	-0.025	0.003	1.98E-13	6	0.373	Y	Y	34594039
rs11669971	19	53400107	*ZNF320*	intronic	C	G	202406	0.690	0.022	0.004	6.92E-09	39.3	0.159	Y	Y[a]	29403010; 35710981
rs6026578	20	57463472	*LOC101927932*	ncRNA_intronic	C	G	202406	0.279	-0.027	0.004	9.42E-14	0	0.520	Y	Y	29403010; 34594039
rs6061532	20	61018541	*MIR1-1HG-AS1*	ncRNA_intronic	T	C	202406	0.606	0.019	0.003	2.41E-08	0	0.697	Y	Y[a]	29403010; 34226706
rs128494	21	37834258	*CLDN14*	intronic	T	C	202406	0.571	-0.020	0.003	7.26E-10	25.9	0.249	Y	Y	29403010; 31015462
rs59315374	22	24188193	*DERL3, SLC2A11*	intergenic	C	G	197904	0.700	-0.024	0.004	2.57E-09	54.2	0.088	Y	Y[a]	29403010; 35710981

rs6001939	22	40892794	*MRTFA*	intronic	T	C	202406	0.276	0.034	0.004	7.42E-19	0	0.854	Y	Y[a]	29403010;30604766
rs2143919	22	43114040	*A4GALT*	intronic	C	G	202406	0.799	-0.023	0.004	1.24E-08	0	0.711	Y	Y	29403010;31451708

eGFR, estimated glomerular filtration rate; GWAS, genome-wide association study.

[a] a different SNP in the same genomic locus.

eTable 3. Previously reported variants significantly associated with SCr ($P < 5\times10^{-8}$) in the GWAS meta-analyses of kidney functions in Japanese

rsID	Chr	Position	Gene	Function	Effect allele	Other allele	N	EAF	Beta	SE	*P*	I2	HetPVal	Reported in JP/EAS	Reported in CEU/TE	PMID
rs491334	1	10750919	*CASZ1*	intronic	A	C	200845	0.134	0.030	0.005	3.19E-09	0	0.695	Y	Y[a]	34594039
rs848301	1	16315262	*ZBTB17*, *SRARP*	intergenic	C	G	200845	0.333	0.027	0.004	1.17E-13	0	0.429	Y	Y[a]	20604766; 34594039; 29403010
rs10917375	1	23708989	*TCEA3*	intronic	T	C	200845	0.788	0.032	0.004	5.15E-16	59.8	0.041	Y	Y[a]	34594039; 33462484
rs10889348	1	63078222	*DOCK7*	intronic	A	T	200845	0.863	0.026	0.005	4.55E-08	0	0.458	Y	Y[a]	35213538
rs278853	1	78130243	*ZZZ3*	intronic	T	C	200845	0.398	-0.025	0.003	5.59E-14	69	0.012	Y[a]	Y[a]	36329257
rs2990246	1	155197602	*GBAP1*	upstream	C	G	200845	0.843	-0.041	0.005	4.92E-18	55.6	0.061	Y	Y[a]	29403010; 31015462
rs34720381	1	171455322	*PRRC2C*	intronic	T	C	200845	0.069	0.037	0.006	7.64E-09	0	0.729	Y	Y	29403010; 31152163

rs3111244	1	211395345	*KCNH1, RCOR3*	intergenic	A	G	200845	0.580	0.019	0.003	1.78E-08	0	0.948	Y	Y	34594039
rs1260326	2	27730940	*GCKR*	exonic	T	C	200845	0.558	-0.039	0.003	1.69E-33	23.9	0.262	Y	Y	27588450; 34594039
rs1527649	2	54581356	*C2orf73*	intronic	T	C	200845	0.743	-0.023	0.004	3.19E-10	38.7	0.163	Y	Y	30604766
rs2118304	2	65681937	*SPRED2, MIR4778*	intergenic	A	G	200845	0.856	-0.029	0.005	2.34E-09	29.1	0.228	Y[a]	Y[a]	35710981
rs11123169	2	113967075	*PSD4*	downstream	T	C	200845	0.731	-0.029	0.004	5.09E-16	0	0.878	Y	Y	29403010; 34272381
rs7596689	2	122039095	*TFCP2L1*	intronic	A	C	200845	0.946	-0.060	0.007	2.34E-17	0	0.826	Y	Y[a]	27588450; 34594039
rs12473366	2	163075264	*FAP*	intronic	A	T	200845	0.871	0.029	0.005	3.62E-09	0	0.928	Y[a]	Y[a]	34594039
rs3770636	2	170202833	*LRP2*	intronic	T	G	200845	0.806	0.059	0.004	5.84E-46	76.3	0.002	Y	Y	31015462
rs3731792	2	176983586	*HOXD10*	intronic	C	G	200845	0.217	-0.023	0.004	1.18E-08	0	0.441	Y[a]	Y[a]	34594039
rs2364730	2	178141063	*NFE2L2, LOC100130691*	intergenic	A	G	200845	0.417	-0.019	0.003	1.03E-08	0	0.503	Y[a]	Y[a]	34594039

rs55932961	2	211549856	*CPS1, ERBB4*	intergenic	A	G	200845	0.156	0.042	0.005	3.42E-20	51.1	0.085	Y	Y	29403010; 34594039
rs7598172	2	217677999	*IGFBP5, TNP1*	intergenic	T	G	200845	0.511	0.031	0.003	7.38E-22	81.9	1.87E-04	Y	Y[a]	29403010; 28452372
rs182545	3	12192525	*SYN2*	intronic	C	G	200845	0.648	-0.020	0.003	1.47E-09	0	0.920	Y[a]	Y[a]	34594039; 31152163
rs7640050	3	38525864	*ACVR2B*	UTR3	A	C	200845	0.658	-0.019	0.003	7.13E-09	0	0.536	Y[a]	Y[a]	36329257
rs9816720	3	121714684	*ILDR1*	intronic	A	G	200845	0.762	0.025	0.004	9.85E-11	0	0.451	Y	Y	34594039
rs6767158	3	141228874	*RASA2*	intronic	T	C	200845	0.341	0.020	0.003	4.03E-09	37.2	0.173	Y	N	34594039
rs57900283	3	169150797	*MECOM*	intronic	T	C	200845	0.278	0.024	0.004	1.83E-11	23.1	0.267	Y	Y[a]	34594039
rs5029970	3	186434491	*KNG1*	upstream	T	C	200845	0.701	-0.024	0.004	5.59E-12	55.4	0.062	Y	Y[a]	29403010; 34594039
rs13108218	4	3443931	*HGFAC*	intronic	A	G	200845	0.546	-0.024	0.003	2.59E-13	16.6	0.309	Y	Y	30604766
rs2279252	4	5800494	*EVC*	exonic	A	G	200845	0.097	-0.035	0.005	6.45E-11	49.6	0.094	Y	Y	35710981

rs10025351	4	77394095	*SHROOM3*	intronic	T	C	200845	0.216	0.050	0.004	1.04E-37	0	0.406	Y	Y[a]	29403010; 31152163
rs16998073	4	81184341	*PRDM8, FGF5*	intergenic	A	T	200845	0.700	0.032	0.004	1.08E-19	1.6	0.397	Y	Y	36329257; 31451708
rs2075633	4	100238998	*ADH1B*	intronic	T	C	200845	0.166	-0.025	0.004	5.54E-09	17.8	0.302	Y[a]	Y[a]	35710981
rs3765637	4	101114121	*LOC101929353*	ncRNA_splicing	A	T	200845	0.537	-0.022	0.003	1.49E-11	0	0.658	Y[a]	Y[a]	29403010; 35710981
rs223413	4	103732866	*UBE2D3*	intronic	T	C	200845	0.357	-0.019	0.003	1.49E-08	0	0.750	Y	Y	30604766
rs13159523	5	676962	*TPPP*	intronic	A	G	200845	0.431	0.021	0.003	1.24E-10	61.5	0.034	Y	Y	31152163
rs7714709	5	34510387	*NONE, RAI14*	intergenic	A	G	200845	0.740	-0.029	0.004	8.52E-14	0	0.730	Y	Y[a]	29403010
rs72753700	5	44117677	*NNT, FGF10*	intergenic	A	G	200845	0.246	0.025	0.004	5.57E-11	42.7	0.137	Y[a]	Y[a]	31152163
rs11742501	5	151107096	*CLMAT3, ATOX1*	intergenic	T	C	200845	0.906	0.037	0.006	1.56E-11	0	0.640	Y	N	29403010
rs3812036	5	176813404	*SLC34A1*	intronic	T	C	200845	0.251	0.037	0.004	8.81E-24	57.8	0.050	Y	Y	29403010; 28452372

rs146663041	6	29034979	*LOC100129636*	ncRNA_intronic	T	G	200845	0.929	-0.039	0.007	1.36E-08	71.2	0.008	Y	N	30604766
rs6935129	6	34371707	*RPS10-NUDT3*	intronic	A	G	200845	0.558	0.021	0.003	4.09E-10	0	0.805	Y	Y	29403010; 30604766
rs35985639	6	41673580	*TFEB*	intronic	A	G	200845	0.272	-0.026	0.004	4.92E-12	0	0.941	Y	Y[a]	29403010; 33462484
rs881858	6	43806609	*VEGFA, LINC02537*	intergenic	A	G	200845	0.878	0.042	0.005	9.64E-18	80	5.06E-04	Y	Y	27588450
rs2143081	6	50782834	*TFAP2D, TFAP2B*	intergenic	A	G	200845	0.576	-0.018	0.003	2.42E-08	0	0.453	Y	Y	35710981
rs4715517	6	54973761	*HCRTR2*	intronic	A	C	58748	0.085	0.116	0.011	4.94E-24	16.3	0.310	Y	Y	34594039
rs6907843	6	90109374	*RRAGD*	intronic	T	C	200845	0.084	0.036	0.006	1.49E-09	0	0.533	Y	Y	34594039
rs241812	6	100890991	*SIM1*	intronic	A	G	200845	0.430	0.018	0.003	1.14E-08	0	0.471	Y	Y[a]	29403010; 34594039
rs7766720	6	107172979	*LINC02532*	ncRNA_intronic	T	C	200845	0.819	-0.028	0.004	1.54E-10	27.6	0.238	Y	Y	31152163
rs2781656	6	131882571	*AKAP7, ARG1*	intergenic	T	C	200845	0.321	0.026	0.003	8.16E-14	15.6	0.315	Y	Y[a]	29403010
rs62430664	6	133842133	*TARID*	ncRNA_intronic	T	C	58748	0.907	0.057	0.010	3.51E-08	0	0.517	Y[a]	Y[a]	33462484;

																35710981
rs533452	6	160697762	*SLC22A2, SLC22A3*	intergenic	A	G	196343	0.108	-0.045	0.005	1.23E-17	49.4	0.115	Y[a]	Y[a]	29403010; 28452372
rs10277115	7	1285195	*UNCX, MICALL2*	intergenic	A	T	200845	0.683	-0.055	0.004	9.44E-56	42.8	0.136	Y	Y	22797727; 26831199
rs78503047	7	41068572	*LINC01450, LINC01449*	intergenic	T	C	200845	0.117	0.037	0.005	1.88E-13	29.1	0.228	Y	Y	31152163
rs6465825	7	77416439	*RSBN1L, TMEM60*	intergenic	T	C	200845	0.768	-0.023	0.004	8.09E-10	0	0.510	Y	Y	26831199
rs3807121	7	129505832	*UBE2H*	intronic	A	G	200845	0.768	0.021	0.004	3.41E-08	0	0.486	Y[a]	Y[a]	31152163
rs868822	7	156252939	*LOC285889, LINC01006*	intergenic	T	G	200845	0.500	-0.017	0.003	4.05E-08	61	0.036	Y	Y	31152163
rs11135780	8	23746429	*STC1, ADAM28*	intergenic	A	G	200845	0.813	-0.032	0.004	3.08E-14	0	0.799	Y[a]	Y[a]	35710981
rs4422737	8	32399849	*NRG1*	intronic	A	G	200845	0.832	-0.030	0.004	3.55E-12	56.8	0.055	Y	Y	34594039
rs6982502	8	126479362	*TRIB1, LINC00861*	intergenic	T	C	200845	0.564	0.019	0.003	5.60E-09	0	0.422	Y[a]	Y[a]	34594039
rs75834729	8	130371638	*CCDC26*	ncRNA_intronic	T	C	200845	0.287	-0.020	0.004	1.28E-08	57.2	0.053	Y	Y[a]	29403010

rs62555928	9	33882692	*UBE2R2*	intronic	T	C	200845	0.510	-0.021	0.003	6.67E-11	0	0.812	Y[a]	Y[a]	34272381
rs4744712	9	71434707	*PIP5K1B*	intronic	A	C	200845	0.380	0.020	0.003	1.52E-09	54.9	0.064	Y	Y	28452372; 27588450
rs10156580	9	130686549	*PIP5KL1*	intronic	A	G	200845	0.723	0.021	0.004	1.86E-08	25.2	0.254	Y[a]	Y[a]	34272381
rs9411378	9	136145425	*ABO*	intronic	A	C	200845	0.260	-0.026	0.004	2.19E-11	14.2	0.324	Y[a]	Y[a]	34594039
rs75048142	9	140162664	*NELFB*	intronic	T	G	200845	0.092	0.033	0.006	3.69E-08	34.7	0.190	Y[a]	Y[a]	35710981
rs80282103	10	899071	*LARP4B*	intronic	A	T	200845	0.923	-0.047	0.006	4.76E-14	66.8	0.017	Y	Y	31152163; 28452372
rs12785213	10	69964674	*MYPN*	intronic	A	G	200845	0.324	-0.033	0.003	1.92E-21	0	0.837	Y[a]	Y[a]	29403010; 31152163
rs12240570	10	75016364	*DNAJC9-AS1*	ncRNA_intronic	A	T	200845	0.244	0.024	0.004	1.44E-09	0	0.554	Y[a]	Y[a]	34594039
rs10748830	10	104548382	*WBP1L*	intronic	A	C	200845	0.657	-0.021	0.003	1.12E-09	0	0.513	Y[a]	Y[a]	30604766
rs10901809	10	126410804	*FAM53B*	intronic	T	C	200845	0.289	-0.024	0.004	7.41E-12	15.2	0.318	Y[a]	Y[a]	30604766

rs62650851	11	2148166	*H19, IGF2*	intergenic	C	G	200845	0.434	-0.025	0.003	4.86E-15	0	0.548	Y	Y	29403010; 35710981
rs2220970	11	9857749	*SBF2*	intronic	A	G	200845	0.340	0.020	0.003	1.51E-09	0	0.475	Y[a]	Y[a]	35710981
rs963837	11	30749090	*MPPED2, DCDC1*	intergenic	T	C	200845	0.651	0.045	0.004	7.40E-37	25.4	0.252	Y	Y	29403010; 30604766
rs61897431	11	47427667	*MIR4487, SLC39A13*	intergenic	T	C	200845	0.742	-0.024	0.004	1.14E-10	0	0.524	Y	Y	31152163
rs7123489	11	65524252	*RNASEH2C, AP5B1*	intergenic	A	C	200845	0.161	0.032	0.004	6.81E-13	0	0.894	Y	Y	31015462
rs7940841	11	78079084	*GAB2*	intronic	A	G	200845	0.600	0.028	0.004	1.68E-14	21.7	0.277	Y	Y	29403010; 31152163
rs11062070	12	343632	*SLC6A13*	intronic	T	C	200845	0.412	0.023	0.003	1.28E-11	7.9	0.361	Y	Y[a]	29403010
rs632887	12	3392351	*TSPAN9*	UTR3	A	G	200845	0.709	-0.026	0.004	4.95E-13	0	0.468	Y	Y	30604766
rs7302787	12	15344066	*RERG*	intronic	T	G	200845	0.358	-0.023	0.003	2.48E-11	0	0.526	Y	Y	31451708
rs4399402	12	23899593	*SOX5*	intronic	A	G	200845	0.183	0.023	0.004	2.78E-08	22.9	0.269	Y	Y[a]	29403010; 35710981

rs1275609	12	76271183	*KRR1, PHLDA1*	intergenic	A	G	196343	0.597	-0.026	0.004	1.16E-13	0	0.637	Y	Y	29403010; 30604766
rs79105258	12	111718231	*CUX2*	intronic	A	C	200845	0.244	0.053	0.004	4.69E-40	54.7	0.066	Y	Y	29403010; 30604766
rs67332916	13	42749711	*DGKH*	intronic	T	C	200845	0.404	-0.026	0.003	4.66E-15	0	0.685	Y	Y[a]	29403010
rs55633570	13	48564746	*SUCLA2*	intronic	A	G	200845	0.611	-0.018	0.003	3.42E-08	38.6	0.164	Y	N	35710981; 34594039
rs478141	13	72346092	*DACH1*	intronic	A	G	200845	0.848	-0.033	0.004	1.62E-13	0	0.686	Y[a]	Y[a]	29403010; 26831199
rs61989532	14	37568235	*SLC25A21*	intronic	T	C	200845	0.112	0.030	0.005	9.03E-09	0	0.918	Y	N	34594039
rs11850798	14	50578362	*VCPKMT*	intronic	A	G	200845	0.442	-0.018	0.003	3.14E-08	0	0.805	Y	N	34594039
rs7168414	15	45639612	*LOC101928414, GATM*	intergenic	T	C	200845	0.841	0.029	0.005	1.09E-09	40.6	0.151	Y[a]	Y[a]	31152163
rs28441244	15	50729642	*USP8*	intronic	T	C	200845	0.819	0.031	0.005	2.30E-11	45.1	0.121	Y	Y	34594039
rs17730281	15	53907948	*WDR72*	exonic	A	G	200845	0.395	-0.043	0.003	1.12E-38	0	0.570	Y	Y	34594039

rs12148134	15	67592098	*IQCH*	intronic	C	G	200845	0.417	0.020	0.004	3.33E-08	7.1	0.366	Y[a]	Y[a]	31015462
rs11636251	15	76239020	*NRG4*	intronic	T	C	200845	0.501	0.031	0.004	2.21E-18	35.7	0.183	Y	Y	30604766
rs16972495	15	81148739	*CEMIP*	intronic	A	C	200845	0.748	0.025	0.004	3.19E-11	0	0.609	Y	Y	29403010; 33462484
rs148840914	15	99306048	*IGF1R*	intronic	A	G	200845	0.266	0.034	0.004	1.34E-20	29.1	0.227	Y[a]	Y[a]	29403010; 33462484
rs35208507	16	20388929	*PDILT*	intronic	A	G	200845	0.779	0.064	0.004	1.90E-61	0	0.483	Y	Y[a]	34594039
rs7186298	16	21088031	*DNAH3*	intronic	T	C	200845	0.475	0.020	0.003	9.90E-10	0	0.640	Y[a]	Y[a]	35710981
rs12935539	16	51754991	*SALL1, LINC01571*	intergenic	T	C	200845	0.685	-0.025	0.004	2.91E-13	34.9	0.189	Y	Y	29403010; 30604766
rs12444699	16	69823443	*WWP2*	intronic	A	G	200845	0.870	0.032	0.005	1.65E-09	63.4	0.027	Y[a]	Y[a]	31152163
rs216195	17	2203167	*SMG6*	exonic	T	G	200845	0.338	0.019	0.003	2.53E-08	14	0.325	Y[a]	Y[a]	29403010; 35710981
rs7222869	17	19417761	*RNF112, SLC47A1*	intergenic	A	G	200845	0.936	-0.042	0.007	1.23E-09	0	0.509	Y	Y[a]	33462484; 29403010

rs7212715	17	37607904	*MED1*	upstream	T	C	200845	0.766	0.035	0.004	1.10E-16	0	0.944	Y	Y[a]	29403010; 34272381
rs9895661	17	59456589	*BCAS3*	intronic	T	C	200845	0.470	-0.054	0.004	3.46E-54	87.1	2.99E-06	Y	Y	28452372; 27588450
rs16942751	18	24393213	*PCAT18, AQP4*	intergenic	A	C	200845	0.298	0.024	0.004	5.51E-12	0	0.983	Y	Y	29403010; 30604766
rs2337106	18	46460903	*SMAD7*	intronic	C	G	200845	0.471	0.023	0.003	1.44E-12	8.8	0.356	Y	Y	30604766
rs549752	18	77158225	*NFATC1*	intronic	A	G	200845	0.318	0.046	0.004	3.21E-40	18.1	0.300	Y	Y[a]	28452372
rs2241359	19	14586245	*PTGER1*	upstream	A	G	200845	0.689	0.020	0.003	4.16E-09	0	0.779	Y[a]	Y[a]	35710981
rs8101881	19	33364628	*SLC7A9, CEP89*	intergenic	T	C	200845	0.337	0.025	0.003	5.35E-14	0	0.532	Y	Y	34594039
rs3814995	19	36342212	*NPHS1*	exonic	T	C	200845	0.614	-0.018	0.003	3.53E-08	0	0.760	Y	Y	35710981
rs6026578	20	57463472	*LOC101927932*	ncRNA_intronic	C	G	200845	0.279	0.026	0.004	3.81E-13	15.8	0.314	Y	Y	29403010; 34594039
rs128494	21	37834258	*CLDN14*	intronic	T	C	200845	0.571	0.019	0.003	4.96E-09	6.8	0.368	Y	Y	29403010; 31015462

GWAS, genome-wide association study; SCr, serum creatinine.

[a] a different SNP in the same genomic locus.

eTable 4. Independent signals significantly associated with eGFR ($P<5\times10^{-8}$) in the GWAS meta-analyses of kidney functions in Japanese

GenomicLocus No	rsID	Lead SNP	Chr	Position	Gene	Function	Effect allele	Other allele	Original					Conditional analysis		
									N	EAF	Beta	SE	*P*	Beta	SE	*P*
1	rs13086653	No	3	168738496	*LINC02082, MECOM*	intergenic	A	G	202406	0.907	0.036	0.006	1.17E-09	0.038	0.006	1.34E-10
1	rs16853722	Yes	3	169150632	*MECOM*	intronic	T	C	202406	0.722	0.027	0.004	4.38E-13	0.028	0.004	5.10E-14
2	rs9368805	Yes	6	34225075	*SMIM29, NUDT3*	intergenic	T	C	202406	0.218	-0.026	0.004	6.00E-10	-0.025	0.004	1.87E-09
2	rs6935129	No	6	34371707	*RPS10-NUDT3*	intronic	A	G	202406	0.558	-0.021	0.003	1.65E-09	-0.020	0.003	5.13E-09
3	rs61008480	No	6	53925808	*MLIP*	intronic	T	C	143658	0.028	-0.084	0.013	4.01E-10	-0.092	0.013	6.04E-12
3	rs142516820	No	6	54003052	*MLIP*	exonic	A	G	202406	0.977	0.100	0.012	4.68E-18	0.068	0.012	7.58E-09
3	rs1508631	No	6	54285667	*TINAG, FAM83B*	intergenic	A	T	202406	0.307	0.024	0.004	1.79E-11	0.022	0.004	2.65E-09
3	rs4715517	Yes	6	54973761	*HCRTR2*	intronic	A	C	58748	0.085	-0.117	0.012	1.98E-24	-0.088	0.012	1.43E-13

3	rs9370390	No	6	55013848	*HCRTR2*	intronic	A	G	202406	0.244	-0.035	0.004	1.75E-16	-0.024	0.004	1.54E-08
4	rs75397777	No	6	160601711	*SLC22A1, SLC22A2*	intergenic	A	G	54246	0.023	-0.147	0.023	8.52E-11	-0.151	0.023	2.50E-11
4	rs10945658	No	6	160658130	*SLC22A2*	intronic	T	C	197904	0.059	-0.050	0.007	1.12E-12	-0.046	0.007	6.55E-11
4	rs533452	Yes	6	160697762	*SLC22A2, SLC22A3*	intergenic	A	G	197904	0.108	0.045	0.005	1.07E-17	0.043	0.005	4.60E-16
5	rs7222869	Yes	17	19417761	*RNF112, SLC47A1*	intergenic	A	G	202406	0.936	0.041	0.007	4.32E-09	0.041	0.007	3.09E-09
5	rs116531123	No	17	19491425	*SLC47A1, ALDH3A2*	intergenic	A	C	58748	0.983	0.155	0.027	1.71E-08	0.156	0.027	1.24E-08
6	rs8080123	No	17	59242914	*BCAS3*	intronic	T	G	202406	0.196	0.040	0.004	3.28E-20	0.041	0.004	1.67E-21
6	rs9895661	Yes	17	59456589	*BCAS3*	intronic	T	C	202406	0.469	0.059	0.004	9.01E-60	0.059	0.004	5.66E-61

EAF, effect allele frequency; eGFR, estimated glomerular filtration rate; GWAS, genome-wide association study; SE, standard error; SNP, single-nucleotide polymorphism.

eTable 5. Independent signals significantly associated with SCr ($P<5\times10^{-8}$) in the GWAS meta-analyses of kidney functions in Japanese

GenomicLocus No	rsID	Lead SNP	Chr	Position	Gene	Function	Effect allele	Other allele	Original					Conditional analysis		
									N	EAF	Beta	SE	*P*	Beta	SE	*P*
1	rs6767158	Yes	3	141228874	*RASA2*	intronic	T	C	200845	0.341	0.020	0.003	5.76E-09	0.021	0.003	8.95E-10
1	rs9942075	No	3	141722878	*TFDP2*	intronic	A	T	200845	0.254	-0.021	0.004	3.80E-08	-0.022	0.004	5.86E-09
2	rs13086653	No	3	168738496	*LINC02082, MECOM*	intergenic	A	G	200845	0.907	-0.032	0.006	2.56E-08	-0.034	0.006	4.17E-09
2	rs57900283	Yes	3	169150797	*MECOM*	intronic	T	C	200845	0.278	0.024	0.004	2.62E-11	0.025	0.004	4.33E-12
3	rs6922699	No	6	34235225	*SMIM29, NUDT3*	intergenic	T	C	200845	0.807	-0.023	0.004	2.33E-08	-0.022	0.004	4.61E-08
3	rs6935129	Yes	6	34371707	*RPS10-NUDT3*	intronic	A	G	200845	0.558	0.021	0.003	5.23E-10	0.020	0.003	1.03E-09
4	rs61008480	No	6	53925808	*MLIP*	intronic	T	C	142097	0.028	0.083	0.013	1.55E-10	0.085	0.013	5.46E-11
4	rs142516820	No	6	54003052	*MLIP*	exonic	A	G	200845	0.977	-0.102	0.011	1.08E-19	-0.084	0.011	1.47E-13
4	rs4715517	Yes	6	54973761	*HCRTR2*	intronic	A	C	58748	0.085	0.116	0.011	4.00E-24	0.101	0.012	3.45E-18

5	rs75397777	No	6	160601711	*SLC22A1, SLC22A2*	intergenic	A	G	54246	0.023	0.135	0.023	1.97E-09	0.139	0.023	6.81E-10
5	rs10945658	No	6	160658130	*SLC22A2*	intronic	T	C	196343	0.059	0.042	0.007	1.15E-09	0.038	0.007	3.82E-08
5	rs533452	Yes	6	160697762	*SLC22A2, SLC22A3*	intergenic	A	G	196343	0.108	-0.045	0.005	1.15E-17	-0.043	0.005	2.65E-16
6	rs77829123	No	11	1979861	*MRPL23, MRPL23-AS1*	intergenic	A	G	200845	0.083	0.033	0.006	4.17E-08	0.035	0.006	9.03E-09
6	rs62650851	Yes	11	2148166	*H19, IGF2*	intergenic	C	G	200845	0.434	-0.025	0.003	3.41E-15	-0.026	0.003	7.58E-16
7	rs541940	No	11	30492836	*MPPED2*	intronic	T	C	200845	0.546	0.022	0.003	1.48E-11	0.020	0.003	6.88E-10
7	rs963837	Yes	11	30749090	*MPPED2, DCDC1*	intergenic	T	C	200845	0.651	0.045	0.004	1.83E-36	0.044	0.004	8.56E-35
8	rs7222869	Yes	17	19417761	*RNF112, SLC47A1*	intergenic	A	G	200845	0.936	-0.042	0.007	1.51E-09	-0.042	0.007	1.07E-09
8	rs116531123	No	17	19491425	*SLC47A1, ALDH3A2*	intergenic	A	C	58748	0.983	-0.156	0.027	1.15E-08	-0.157	0.027	8.23E-09
9	rs8073894	No	17	59241155	*BCAS3*	intronic	T	C	200845	0.266	-0.032	0.004	1.69E-17	-0.033	0.004	5.18E-19
9	rs9895661	Yes	17	59456589	*BCAS3*	intronic	T	C	200845	0.470	-0.054	0.004	2.78E-54	-0.055	0.004	1.03E-55

EAF, effect allele frequency; GWAS, genome-wide association study; SCr, serum creatinine; SE, standard error; SNP, single-nucleotide polymorphism.

eTable 6. Allele frequency, effect size, and functional annotation for four SNPs with genome-wide or suggestive significance around *CD36* gene

**SNP**	**Chr**	**Position**	**Gene**	**Function**	**Feature**	**Change in DNA sequence**	**Amino acid change**	**SIFT**	**Polyphen2 HVAR**	**Polyphen2 HDIV**	**CADD**	**Alleles**		**1000 Genomes RAF**					**N**	**EAF**	**Beta**	**SE**	***P***	**I^2**	**HetP**
												**Effect**	**Non-effect**	**AFR**	**AMR**	**EAS**	**EUR**	**SAS**							
rs146148222	7	80304855	*CD36*	intronic							5.712	T	G	-	-	0.990	-	-	200845	0.952	0.045	0.008	4.36E-08	70.1	0.010
rs147077527	7	80283169	*CD36*	intronic							3.147	T	C	-	-	0.974	-	-	58748	0.941	0.068	0.013	1.09E-07	25	0.261
rs75326924	7	80286003	*CD36*	exonic	NM_001289911<br>NM_001371079<br>NM_001127443<br>NM_001289909<br>NM_00112744	C40T<br>C166T<br>C268T<br>C268T<br>C268T<br>C268T	P14S<br>P56S<br>P90S<br>P90S<br>P90S<br>P90S	Damaging (0.913)	Probably damaging (0.928)	Probably damaging (0.906)	30	T	C	-	-	0.990	-	-	200845	0.047	-0.043	0.008	1.77E-07	72.8	0.005

					4<br>NM_00<br>128990<br>8<br>NM_00<br>0072<br>NM_00<br>100154<br>7<br>NM_00<br>100154<br>8<br>NM_00<br>137107<br>4<br>NM_00<br>137107<br>5<br>NM_00<br>137107<br>7<br>NM_00<br>137107<br>8	C268T<br>C268T<br>C268T<br>C268T<br>C268T<br>C268T	P90S<br>P90S<br>P90S<br>P90S<br>P90S<br>P90S																
rs143722364	7	80328352	*CD36, SEMA3C*	intergenic				6.899	T	C	-	-	0.987	-	-	200845	0.950	0.043	0.008	1.78E-07	65.5	0.021	

CADD, combined annotation dependent depletion; Chr, chromosome; EAF, effect allele frequency; Beta, regression coefficient; HetP, P-value for heterogeneity; PolyPhen-2, Polymorphism Phenotyping v2 available at http://genetics.bwh.harvard.edu/pph2/; RAF, reference allele frequency; SE standard error.

Nephroseq™

CD36 Expression in Nakagawa CKD Kidney

Discovery Set Group: Chronic Kidney Disease vs. Normal Kidney

eFigure 1

**Under-expression Gene Rank:**	1504 (in top 8%)	**P-value:**	5.57E-8
**Reporter:**	A_24_P925505	**t-Test:**	-6.215
		**Fold Change:**	-2.916

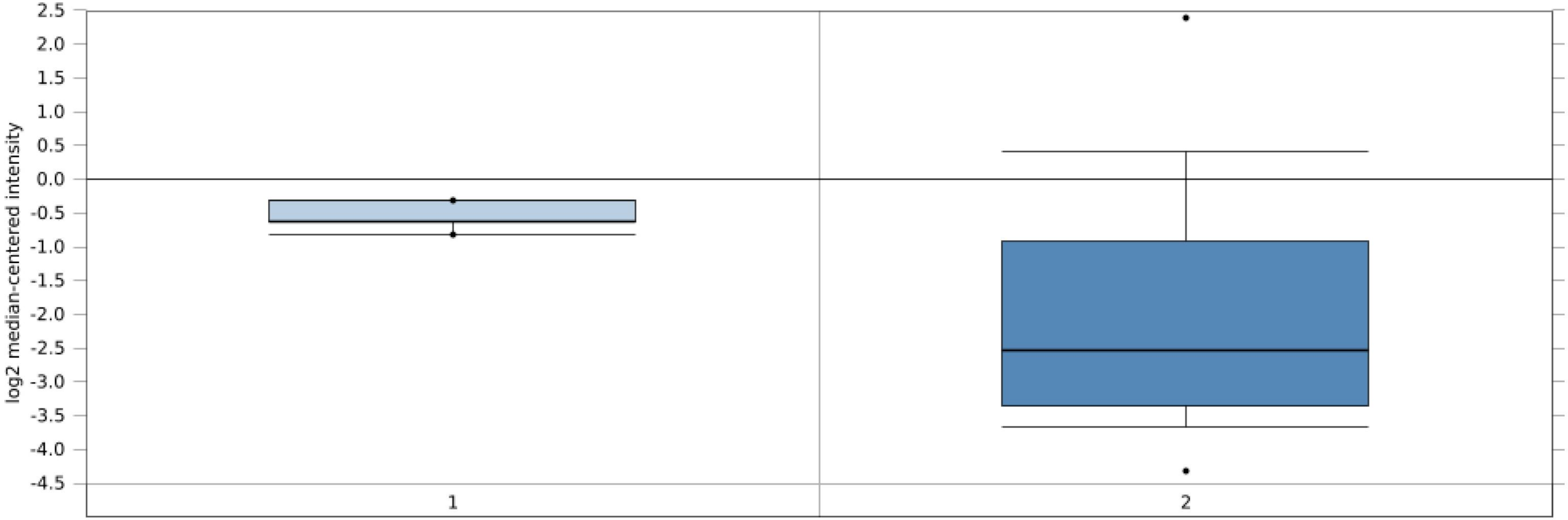

Legend

1. Normal Kidney (5) **2. Chronic Kidney Disease (48)**

Nakagawa CKD Kidney

PLoS One 2015/08/28	61 samples
microarray	Human
Agilent Whole Human Genome Microarray 4x44K (Probe Name Version)	19,063 measured genes
Kidney	

© 2024 The Regents of The University of Michigan. Images from Nephroseq may be used in publications with proper citation. The citation is as follows: Nephroseq (The Regents of The University of Michigan, Ann Arbor, MI) was used for analysis and visualization. For further information, refer to the terms of use.

Nephroseq Source:
http://www.nephroseq.org/resource/main.html#a:1N10846;ac:1N10847,1N10846;d:1N156636784;dso:pvalueUnderex;dt:predefinedClass;ec:[1N2];epv:1N1.1N3,1N21;et:under;f:195259437;g:948;gt:boxplot;p:1N200015275;pg:1;pvf:8206,1N3,1N800094309;scr:datasets;ss:analysis;v:17
